# Testicular ischemia following mesh hernia repair and acute prostatitis

**DOI:** 10.4103/0970-1591.33736

**Published:** 2007

**Authors:** Pepe Pietro, Aragona Francesco

**Affiliations:** Urology Unit, Cannizzaro Hospital, Catania, Italy

**Keywords:** Acute scrotum, inguinal hernia repair, prostatitis, testicular ischemia

## Abstract

We present a case of a man admitted to our Hospital for right acute scrotum that six months before had undergone a right hernioplasty with mesh implantation. Clinical history and testicular color Doppler sonography (CDS) patterns suggested an orchiepididymitis following acute prostatitis. After 48h the clinical picture worsened and testicular CDS showed a decreased telediastolic velocity that suggested testicular ischemia. The patient underwent surgical exploration: spermatic cord appeared stretched by an inflammatory tissue in absence of torsion and releasing of spermatic cord was performed.

In patients with genitourinary infection who previously underwent inguinal mesh implantation, testicular CDS follow-up is mandatory.

## INTRODUCTION

The acute scrotum constitutes the most common urological emergency and color Doppler sonography (CDS), along with the physical exam, represents the imaging modality more frequently employed in the clinical assessment of acute scrotum.[1]

The two most important entities that must be ruled out in every case of acute scrotal pain are torsion of spermatic cord and orchiepididymitis, while other causes occur more rarely. In case of inguinal hernia repair using mesh techniques the spermatic cord is potentially affected by chronic inflammatory tissue remodeling that may impair testicular perfusion inducing acute scrotum.[2]

A rare case of testicular ischemia following mesh hernia repair and acute prostatitis that presented as acute scrotum is reported herein.

## CASE REPORT

**Figure 1:**
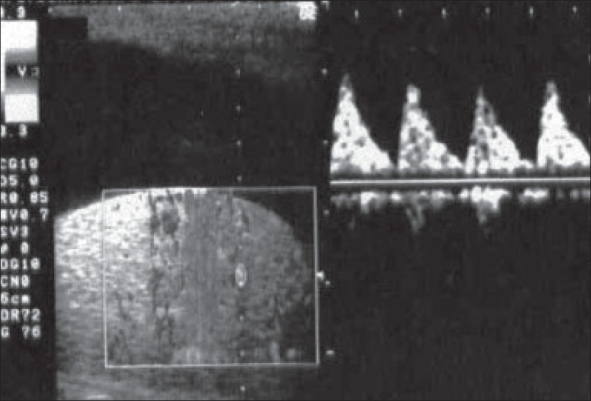
Absence of TDV on intratesticular artery

A 23-year-old man was admitted for right orchialgia arising 24h before admission; although he referred urinary symptoms (stranguria, pollakiuria and gross hematuria) and fever of five days’ duration, no therapy was previously administered. Six months before, the patient underwent a mesh implantation to treat a recurrent right inguinal hernia. At admission, history, genitourinary exam, blood and urine test and transrectal CDS suggested an acute prostatitis; testicular CDS revealed normal signal in correspondence of the testis and epididymis. The patient was hospitalized and submitted to antibiotic therapy. After 48h the clinical picture worsened: testicular pain and fever increased, CDS showed a decreased TDV (telediastolic velocity) on intratesticular artery [Figure 1] and a high SPV (systolic peak velocity) in correspondence of the spermatic cord near the external abdominal ring [Figure 2a]. This sonographic pattern highly suggested a testicular ischemia[3] and the patient underwent surgical exploration: spermatic cord appeared congested, increased in size, compressed and stretched by an inflammatory and scar tissue in the absence of any spermatic cord torsion. Resection of tunica vaginalis and releasing of spermatic cord was performed. After surgery the patient became asymptomatic, CDS returned to a normal pattern [Figure 2b] and antibiotic therapy was administered for two weeks. Histological examination of the tissue surrounding the spermatic cord showed a no specific inflammatory pattern.

**Figure 2a:**
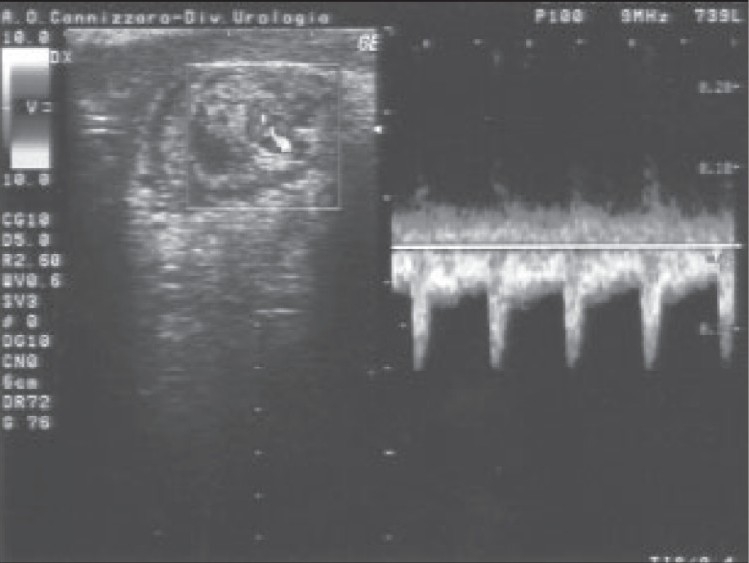
Before surgery: Spiral course of spermatic cord in presence of a high SPV

**Figure 2b:**
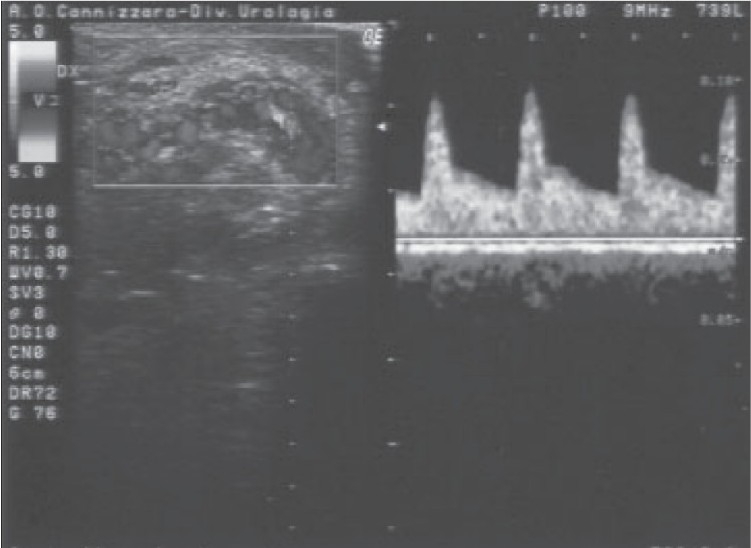
After surgery: Spermatic cord has a linear course with normal CDS parameters

## DISCUSSION

Spermatic cord torsion, orchiepididymitis and trauma constitute the main causes of acute scrotum, although the differential diagnosis refers essentially to the torsion of spermatic cord vs. inflammatory lesions. A careful physical exam and anamnesis combined with CDS parameters often address toward the diagnosis of testicular ischemia that, more rarely, can be secondary to severe epididymitis, inguinal hernia repair,[4] spontaneous trombosis of funicular vessels,[5] xanthogranulomatous funiculitis[6] or filarial.[7] Despite the high diagnostic accuracy of CDS, all authors agree that in the presence of clinical suspicion of testicular torsion, surgical exploration is mandatory;[3] false-negative results with CDS are attributed to an incomplete torsion of the spermatic cord when the systolic value is still recorded while the diastolic one is absent or reduced and to the reactive hyperaemia of the tunica vaginalis which is wrongly interpreted as blood flow into the capsular arteries.

The use of mesh during hernia repair is associated with a relative reduction in the risk of hernia recurrence of around 30-50%; however, there is no apparent difference in recurrence between laparoscopic and open mesh methods of hernia repair.[8] Experimental[9] and clinical studies[10] demonstrated that mesh implantation may induce foreign-body response that led to the encasement of spermatic cord by scar tissue. In most cases, testicular perfusion is not compromised, but a concomitant inflammatory process (i.e., funiculitis secondary to prostatitis) may worsen the blood supply leading to acute scrotum. Peiper *et al*. performed an experimental study in 15 adult male pigs that underwent transinguinal preperitoneal implantation of polypropylene mesh and shouldice repair on the contralateral side. The authors reported that mesh repair led to a decrease of arterial perfusion, testicular temperature and seminiferous tubules.[2] Dilek *et al*.[11] reported no statistically significant differences between the preoperative and postoperative CDS parameters (VPS, TDV and resistive index), while Ersin *et al*.[12] showed that testicular blood flow is influenced during laparoscopic inguinal hernia surgery. Mincheff et al.[13] reported a rare case of upper pole testicular infarction after laparoscopic total extraperitoneal repair of indirect inguinal hernia.

In our case report testicular ischemia was secondary to spermatic cord compression by an inflammatory and scar tissue in absence of torsion and only after releasing of spermatic cord the patient became asymptomatic.

In conclusion, in patients with genitourinary infection who previously had an inguinal mesh implantation, clinical observation and CDS follow-up is mandatory to treat in time this unusual complication.[14]
